# Unraveling the link: exploring the causal relationship between diabetes, multiple sclerosis, migraine, and Alzheimer’s disease through Mendelian randomization

**DOI:** 10.3389/fnins.2023.1233601

**Published:** 2023-08-10

**Authors:** Hua Xue, Li Zeng, Shuangjuan Liu

**Affiliations:** ^1^Department of Neurology, Sichuan Taikang Hospital, Chengdu, Sichuan, China; ^2^Department of Respiratory, Affiliated Hospital of Youjiang Medical University for Nationalities, Baise, Guangxi, China; ^3^Department of Neurology, Qionglai People’s Hospital, Chengdu, Sichuan, China

**Keywords:** diabetes mellitus, multiple sclerosis, migraine, Alzheimer’s disease, Mendelian randomization, genome-wide association study

## Abstract

**Introduction:**

Observational studies suggested that diabetes mellitus [type 1 diabetes mellitus (T1DM), type 2 diabetes mellitus (T2DM)], multiple sclerosis (MS), and migraine are associated with Alzheimer’s disease (AD). However, the causal link has not been fully elucidated. Thus, we aim to assess the causal link between T1DM, T2DM, MS, and migraine with the risk of AD using a two-sample Mendelian randomization (MR) study.

**Methods:**

Genetic instruments were identified for AD, T1DM, T2DM, MS, and migraine respectively from genome-wide association study. MR analysis was conducted mainly using the inverse-variance weighted (IVW) method.

**Results:**

The result of IVW method demonstrated that T2DM is causally associated with risk of AD (OR: 1.237, 95% CI: 1.099–1.391, P: 0.0003). According to the IVW method, there is no causal association between TIDM, MS, migraine, and the risk of AD (all *p* value > 0.05). Here we show, there is a causal link between T2DM and the risk of AD.

**Conclusion:**

These findings highlight the significance of active monitoring and prevention of AD in T2DM patients. Further studies are required to actively search for the risk factors of T2DM combined with AD, explore the markers that can predict T2DM combined with AD, and intervene and treat early.

## 1. Introduction

Diabetes mellitus (DM) is one of the most common chronic metabolic disorders affecting multiple systems (Kashyap et al., 2018; Sempere-Bigorra et al., 2021). Multiple sclerosis (MS), migraine, and Alzheimer’s disease (AD) are common neurological disorders, which cause a lot of inconvenience to the lives of patients and cause a heavy social and economic burden (Oh et al., 2018; Burch, 2019; Sheppard and Coleman, 2020). AD is a degenerative disease of the central nervous system characterized by progressive cognitive dysfunction and behavioral impairment that occurs in the elderly and pre-elderly (Zhang et al., 2021). Clinically, it is manifested as memory impairment, aphasia, apraxia, agnosia, visual–spatial ability impairment, abstract thinking and computing power impairment, and personality and behavior changes (Wang et al., 2020; Zhang et al., 2021). Previous epidemiological studies have observed an increased risk of AD in patients with diabetes, migraine, or multiple sclerosis (Morton et al., 2019; Silva et al., 2019; Benedict et al., 2020). However, the strength and significance of the observed associations of DM, migraine, and MS with AD remain controversial.

Diabetes mellitus is a prevalent chronic disease characterized by chronic hyperglycemia resulting from various causes of defective insulin secretion or defective insulin action that leads to abnormal metabolism of glucose, protein, and fat (Lin et al., 2021; Cloete, 2022). Type 1 diabetes mellitus (T1DM) and type 2 diabetes mellitus (T2DM) are common types of DM. T1DM mostly occurs in adolescents, with acute onset, obvious and severe symptoms, and ketoacidosis as the first symptom, while T2DM mostly occurs in adults over 40 years old and the elderly, mostly obese, with hidden onset and not-so-obvious early symptoms (Gillani et al., 2021; Wu and Liang, 2022). It is widely acknowledged that the complications of DM are numerous and complex, not only affecting retinal arteries and leading to kidney damage but also a risk factor for cardiovascular and cerebrovascular diseases (Demir et al., 2021). In recent years, the associations between DM and AD drawn from epidemiological studies have attracted much attention (Silva et al., 2019; Zhang et al., 2021). Animal experiments have found that dementia is closely related to central insulin resistance (IR) in patients with DM (Rojas et al., 2021). The activities of β-secretase and γ-secretase were enhanced after central IR, and amyloid-β (Aβ) was increased. A large number of Aβ degrading enzymes were combined with insulin, which reduced the degradation of Aβ, and the production, clearance, and aggregation of peptides were unbalanced, resulting in Aβ deposition in the brain (Biessels and Despa, 2018). Alasia et al. cultured cerebellar granule cells *in vitro* and confirmed that Aβ deposition is the direct cause of brain cell apoptosis. At the same time, Aβ deposition also produces senile plaques and induces cognitive impairment in the body (Alasia et al., 2012). Disturbances in glucose metabolism have been shown to impact mitochondrial homeostasis in the brain (Cabezas-Opazo et al., 2015). Studies have found that patients with DM exhibit reduced brain glucose metabolism, which is accompanied by altered expression and decreased activity of mitochondrial energy-related proteins (Solanki et al., 2017). Interestingly, similar symptoms have also been observed in fibroblasts and brain tissues of patients with AD (Solanki et al., 2017). Additionally, a clinical study found that C-peptide was associated with poorer executive function in diabetic patients and insulin resistance may worsen prefrontal cortex function (Lesiewska et al., 2022).

Multiple sclerosis (MS) is an immune-mediated chronic inflammatory demyelinating disease of the central nervous system involving the periventricular, proximal cortex, optic nerve, spinal cord, brainstem, and cerebellum, with spatially multiple and temporally multiple lesions (Baecher-Allan et al., 2018). MS is characterized by a wide variety of clinical symptoms. In addition to the common motor, sensory, visual, and autonomic deficits, cognitive impairment is also a common symptom. The prevalence of cognitive impairment in patients with MS varies by age and may be difficult to distinguish from other causes (e.g., AD) in older patients (Luczynski et al., 2019; Benedict et al., 2020). A cohort study testing MS-related cognitive impairment found greater differences between MS patients (n = 66) and healthy controls (*n* = 22) (Taranu et al., 2022). Although the exact pathogenesis of MS with cognitive impairment is still unclear, it is thought to be primarily related to pathological changes in lesioned and normal-appearing white matter, specific neural gray matter structures, and immunological changes, especially to synaptic transmission and plasticity impact (Portaccio and Amato, 2022). The pathological features of MS include white matter lesions characterized by demyelination and inflammation, resulting in axonal injury and progressive degeneration. Demyelination can cause a reduction in the speed and reliability of axonal transmission. Specifically, it leads to a decrease in conduction speed and information processing speed, leading to cognitive impairment (Conti et al., 2021). Furthermore, a study suggested that abnormalities in the Tryptophan-Kynurenine metabolic system were observed in patients with MS and that the Kynurenine metabolite profile may serve as a biomarker for progressive MS (Polyák et al., 2023). However, the Tryptophan-Kynurenine metabolic pathway was considered to be an important pathway of neuronal damage in neurodegenerative diseases and severe brain injuries (Schwarcz et al., 2012; Battaglia et al., 2023).

Migraine is a highly prevalent and disabling neurological disorder that affects approximately 14.4% of the global population (Zhao et al., 2023). Migraine can cause different levels and types of outcomes, including stroke, subclinical cerebrovascular lesions, hypertension, psychiatric disorders (depression, anxiety, bipolar disorder, panic disorder, and suicide), obesity, and restless legs syndrome. A prospective cohort study involving 679 patients, which followed community-dwelling older adults for 5 years, found that migraines were a significant risk factor for AD and all-cause dementia (Morton et al., 2019). More and more studies have concluded that there is a certain degree of cognitive impairment in patients with migraine, and the degree of cognitive impairment is related to the intensity of headaches, the frequency of attacks, and the duration of attacks in patients (Karami et al., 2019; Klan et al., 2019). The pathological mechanism may be a decrease in the basal metabolic rate of the limbic system composed of the hippocampus, cingulate gyrus, hippocampal gyrus, and amygdala in patients with migraine, affecting the cognitive function of the patients (Guo et al., 2022). A study from China found that serum 5-hydroxytryptamine (5-HT) levels increased in patients with migraine, which may be due to impaired oxidative balance and excessive neuronal excitation (Smith and Ebrahim, 2003). 5-HT is released into the blood, which may further damage the blood–brain barrier and affect cognitive function (Lawlor et al., 2008).

In light of the abovementioned studies, we hypothesized that diabetes, multiple sclerosis, and migraine may be potential factors for AD. However, traditional observational studies are susceptible to potential confounders and reverse causality bias, making the inference of causality difficult. Therefore, the potential causal relationship between appellate disease and AD is unclear. Mendelian randomization (MR) is a new epidemiological technique based on whole genome-wide association studies (GWAS). It uses single nucleotide polymorphisms (SNPs) as instrumental variables (IVs) to reveal causal relationships (Li et al., 2022). Compared with cohort studies and other types of studies, MR can effectively reduce bias; genetic variation is randomly transmitted to offspring and remains unchanged after conception, making it less prone to reverse causality and confounding factors (Grover and Sharma, 2022). Hence, we aimed to explore the causal individual links between AD and diabetes, multiple sclerosis, and migraine through MR studies.

## 2. Materials and methods

Mendelian randomization (MR), proposed by the famous statistician Fisher, is a method of causal inference based on genetic variation and is based on the principle of using the effect of randomly assigned genotypes on phenotypes in nature to infer the effect of biological factors on disease (Nikpay et al., 2015). In MR study, researchers analyze genome-wide association studies (GWAS) to find single nucleotide polymorphisms (SNPs) as instrumental variables (IVs), which are associated with biological factors, and then use these IVs to infer the effects of biological factors on disease (Davey Smith and Hemani, 2014). Since genes are randomly assigned and are not affected by confounding factors, the use of genetic variation to study causality can avoid the influence of confounding factors on the results and improve the reliability of causality inference (Chen et al., 2022).

### 2.1. Study design

**Figure 1:**
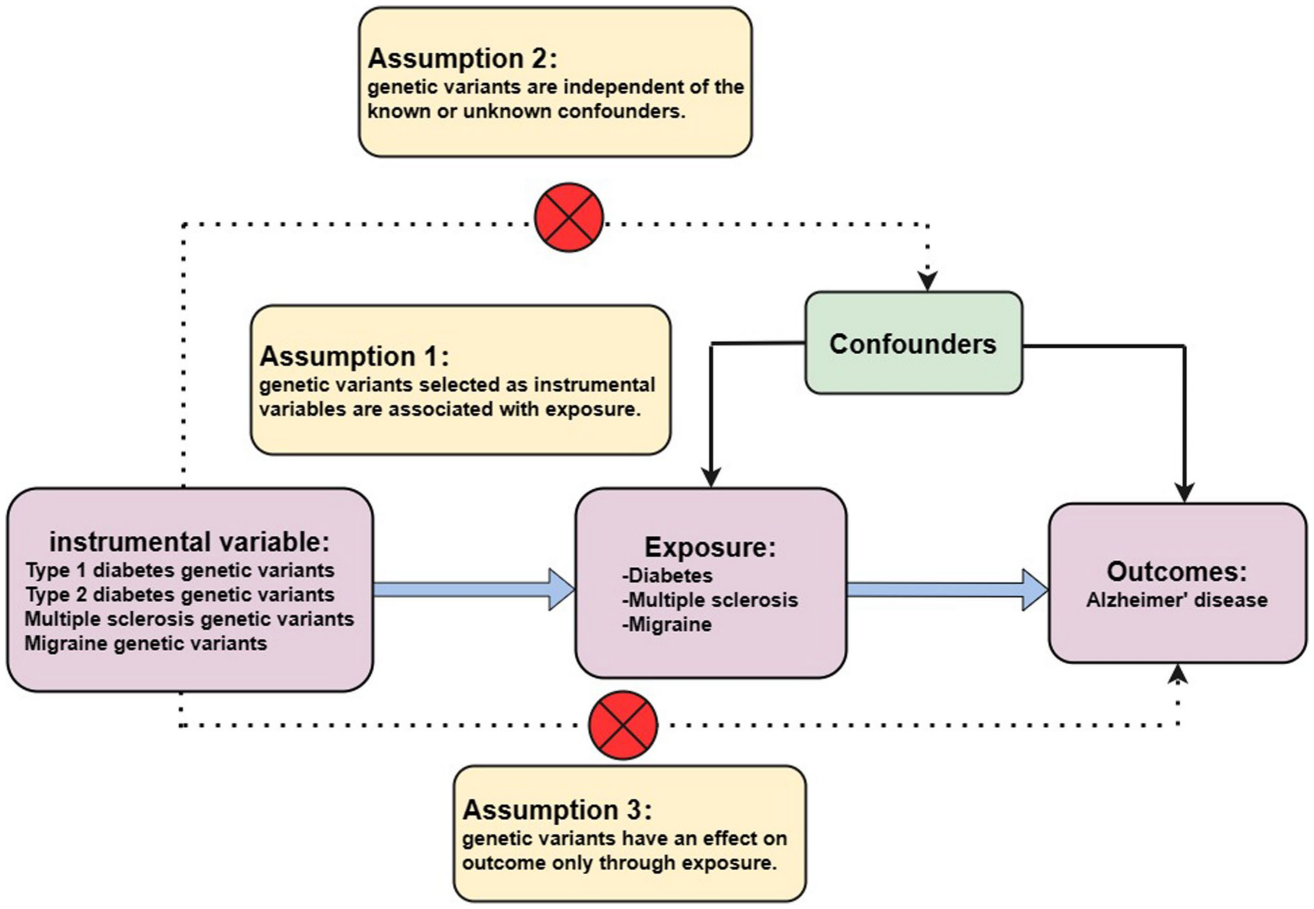
Three assumptions for MR analysis. First, the genetic variants, which are selected as the instrumental variables, are associated with exposure (type 1 diabetes, type 2 diabetes, multiple sclerosis, and migraine). Second, genetic variants are not related to known or unknown confounder factors. Third, genetic variants would have an influence on the outcome (Alzheimer’s disease) only through exposure, not through other pathways.

At present, GWAS has found that hundreds of thousands or even millions of genetic variants are associated with disease outcomes, and these data are the basis of MR analysis (Pierce et al., 2011). In general, MR is based on three conditional assumptions (Figure 1). The first assumption is that the genetic variants chosen as IVs are only associated with exposure (T1DM, T2DM, MS, and migraine) to ensure the relevance of the IVs. Second, selected IVs are uncorrelated with known or unknown confounding factors to ensure the independence of IVs. A final assumption is that genetic variation affects outcomes (AD) only through exposure and not elsewhere to ensure the exclusivity of IVs (Mahajan et al., 2018). Only by satisfying the above three assumptions can the selected IVs make the results as reliable as possible.

### 2.2. Instrument variables selection

We selected SNPs strongly correlated with exposure from GWAS (*p* value <5 × 10^−8^) as IVs at first. The strength of IVs was assessed using the *F*-statistic. The threshold of F-statistic >10 indicated that the genetic variant has a strong estimation effect, in order to effectively avoid the bias caused by IVs (International Multiple Sclerosis Genetics Consortium, 2019). The second step was to remove the SNP linkage disequilibrium (set the parameter as *r^2^* = 0.001, kb = 10,000). Finally, if the selected SNPs were associated with confounder factors that significantly correlated (*p* value <5 × 10^−8^), it was removed from the list of selected SNPs (Hautakangas et al., 2022).

### 2.3. Exposure and outcome GWAS dataset

In this study, the T1DM GWAS summary dataset was derived from a large multigene T1DM genome-wide association meta-analysis that includes 9,266 T1DM cases and 15,575 normal controls (24,840 in total), all from European ancestry (Forgetta et al., 2020). Summary statistics for T2DM have been acquired from the large multigene T2DM genome-wide association meta-analysis that includes 81,412 T2DM cases and 15,575 normal controls (452,244 participants in total) all from European ancestry (Wang et al., 2022). For MS, summary data were extracted from a multiple sclerosis genomic map that involved 47,429 cases (68,374 controls) from the International Multiple Sclerosis Genetics Consortium, 2019; (Park et al., 2023). The summary-level data on migraine was obtained from the largest available genome-wide meta-analysis, comprising 102,084 migraine cases and 771,257 controls， all from European ancestry (Nazarzadeh et al., 2020).

The AD GWAS summary data was downloaded from a large genome-wide analysis involving 21,982 cases (41,944 controls) from the Alzheimer Disease Genetics Consortium (ADGC), which conducted a large-scale meta-analysis in 2019 [Alzheimer Disease Genetics Consortium (ADGC) et al., 2019]. The information on all the genetic datasets used in the current study is displayed in Table 1.

**Table 1:** Detailed information on the studies and datasets used for Mendelian randomization analyses.

Phenotype	Consortium	Ethnicity	Sample size	Cases	Year	PubMed ID
Alzheimer’s disease	PCG	European	63,976	21,982	2019	30820047
Type 1 diabetes	NA	European	24,840	9,266	2020	33830302
Type 2 diabetes	NA	European	452,244	81,412	2018	29632382
Multiple sclerosis	IMSGC	European	115,803	47,429	2019	31604244
Migraine	Neale Lab	European	873,341	102,084	2022	35115687

### 2.4. Statistical analysis

We performed a two-sample MR analysis of the collected GWAS data, using an inverse variance weighting (IVW) approach that combined the Wald ratio as the primary analysis to assess the individual causal association between exposure (TIDM, T2DM, MS, or migraine) and outcome (AD) (Jayaraj et al., 2020). If there was no heterogeneity in the MR results, the fixed effect IVW calculation was used, otherwise, the random effect was performed. IVW is a method for MR to perform a meta-summary on the effect of multiple sites when analyzing multiple SNPs. The premise of the IVW application is that all SNPs are effective IVs and are completely independent of each other (Shen et al., 2022). As a result, the results calculated by the IVW method are more robust and reliable. We first identified genome-wide significant (value of *p* <5 × 10^−8^) and independent (*r^2^* < 0.001, kb = 10,000) SNPs from the exposure dataset as genetic IVs of exposure. The exposure-associated SNPs obtained from the AD dataset were then correlated with the genetics of AD. In addition, additional MR analyses, including MR-Egger regression, weighted median regression (WMR), and Mendelian randomized multivariate residuals and outliers (MR-PRESSO) methods, complement the IVW as these methods can provide more robust estimates in a wider range of scenarios (Jayaraj et al., 2020). MR-Egger regression can provide tests for unbalanced pleiotropy and considerable heterogeneity, whereas larger sample sizes are required for the same underexposed variants. WMR is the median of the distribution function obtained by sorting all individual SNP effect values according to weights. When at least 50% of the information comes from valid IVs, WMR can obtain robust estimates (Tsoporis et al., 2020). In addition, strict instrumental value of *p* thresholds and recalculations were used if results from different MR analyses were inconsistent.

To assess the robustness of the results, we performed a series of sensitivity analyses using Cochran’s Q test, MR-Egger intercept test, and MR-PRESSO global test. All value of *p* of the MR-Egger intercept tests were > 0.05, indicating that no horizontal pleiotropy existed.

All the aforementioned statistical analyses were conducted in “TwoSample MR” (version 0.5.6) and “Mendelian Randomization” (version 0.5.2) packages in the statistical program R (version 4.1.1). Statistical significance was defined as a value of *p* <0.05.

## 3. Results

We obtained 69 SNPs as IVs of T1DM from the GWAS. The F-statistics of 69 SNPs were above the threshold of 10, which indicated that they strongly predicted T1DM in the MR analysis. We obtained 53 genetic variants associated with T2DM as IVs. As for MS, we analyzed 92 genetic variants highly associated with MS as IVs. As for migraine, there were 12 genetic variants associated with migraine as IVs. Detailed information on IVs is shown in Supplementary Tables S1–S4.

**Figure 2:**
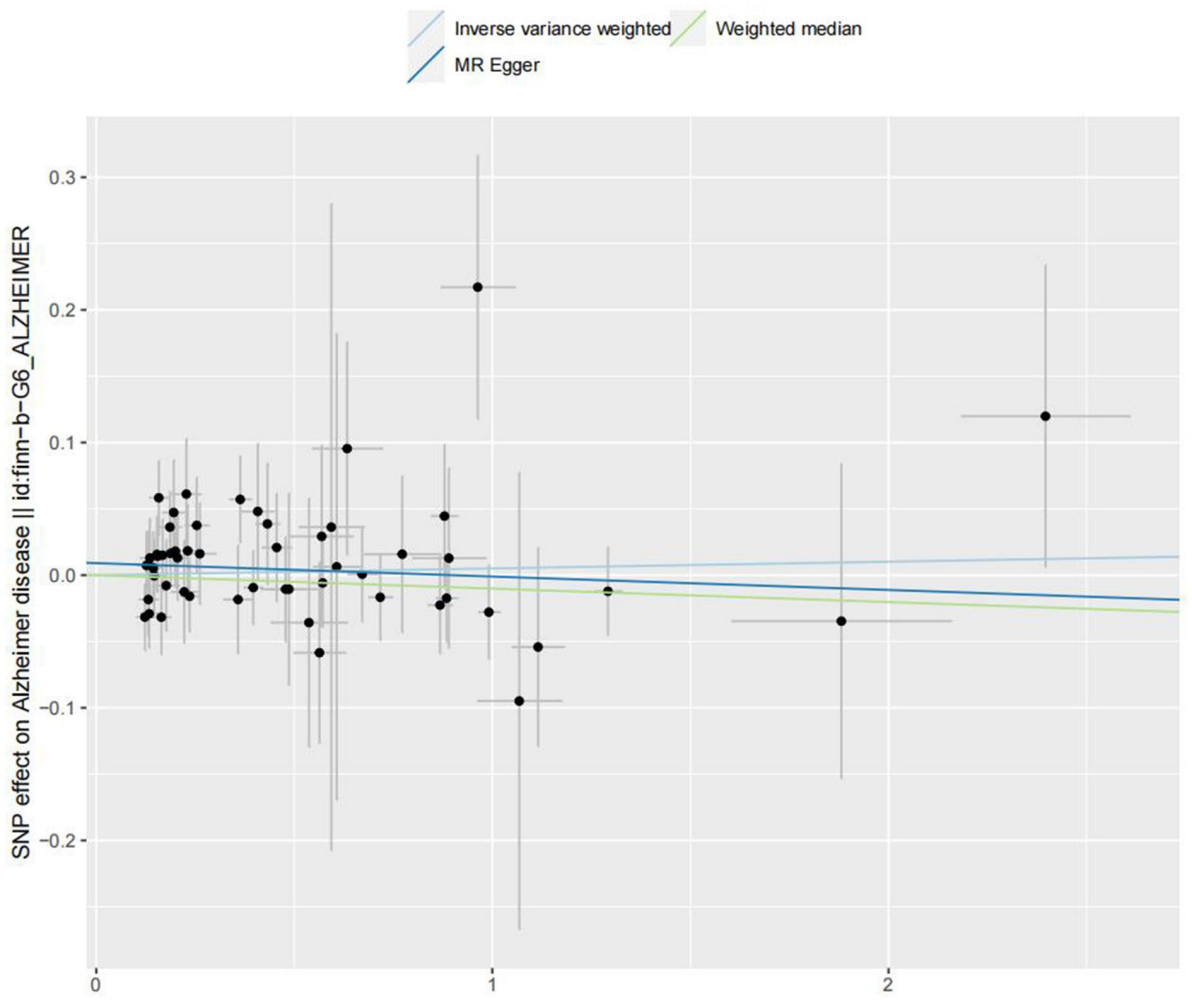
Genetically predicted assessed type 1 diabetes mellitus on Alzheimer’s disease.

**Figure 3:**
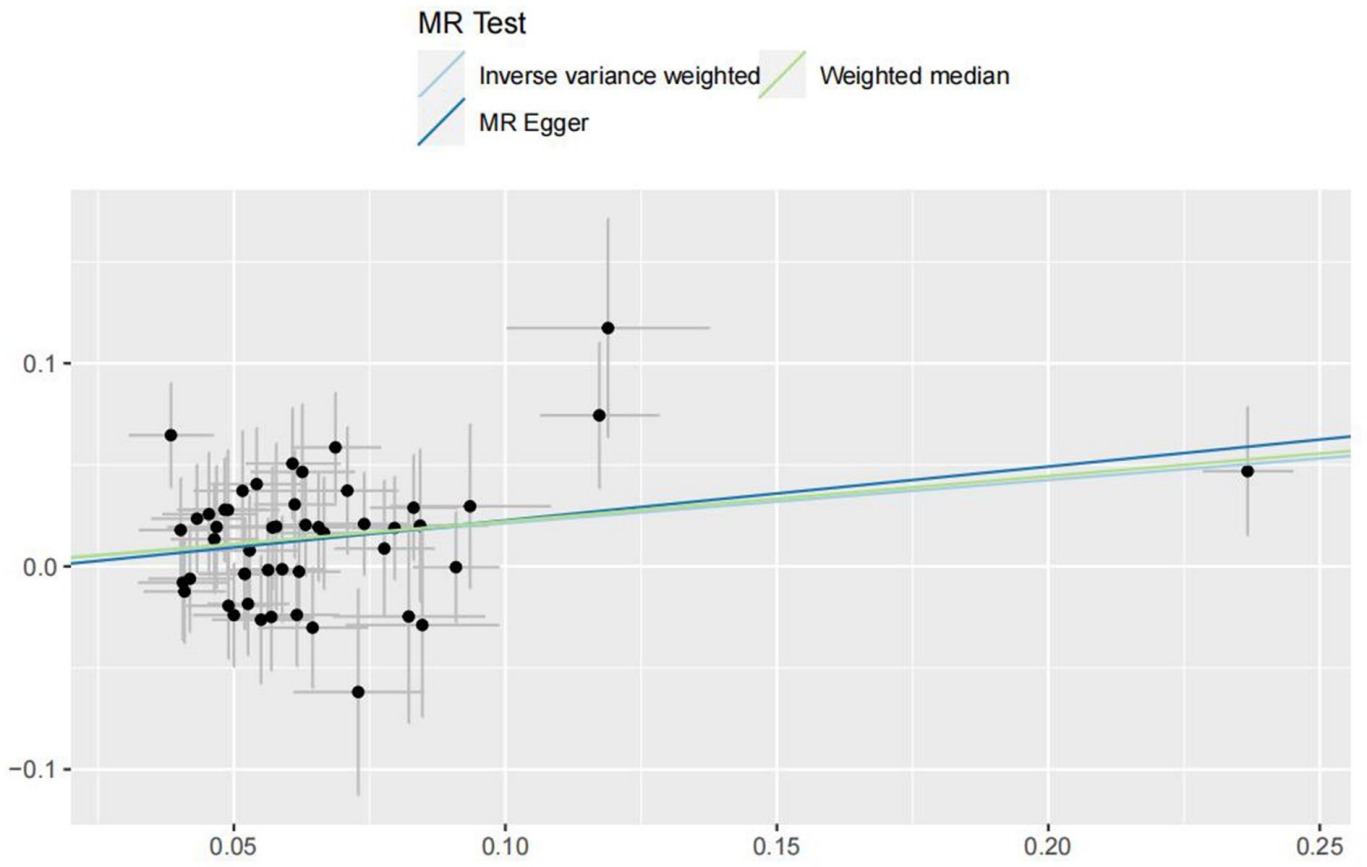
Genetically predicted assessed type 2 diabetes mellitus on Alzheimer’s disease.

**Figure 4:**
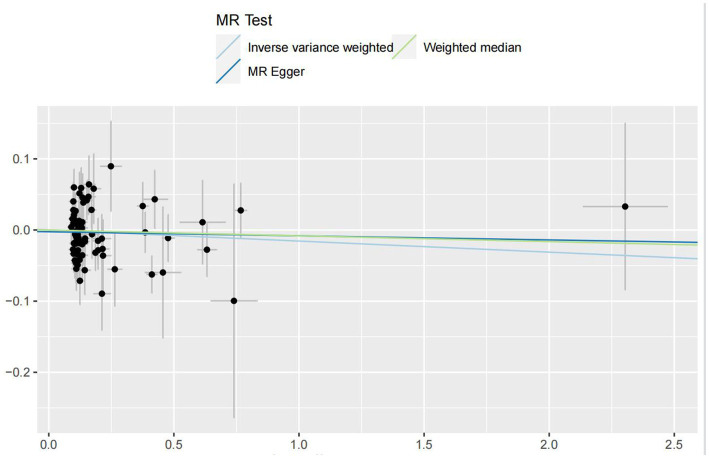
Genetically predicted assessed multiple sclerosis on Alzheimer’s disease.

**Figure 5:**
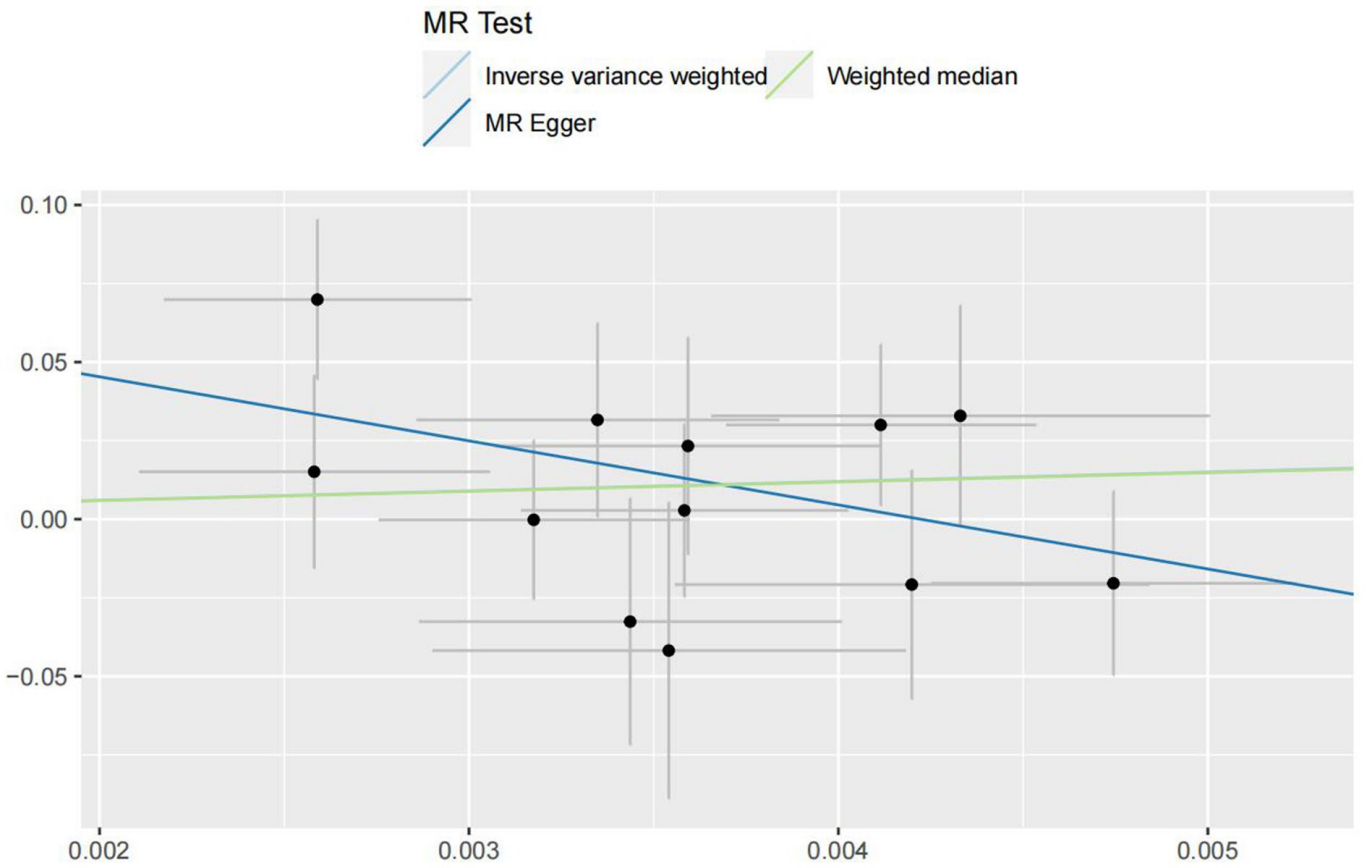
Genetically predicted assessed migraine on Alzheimer’s disease.

In the IVW analyses, no evidence was found for the causal associations between TIDM and the risk of AD (OR: 1.005, 95% CI: 0.983–1.026, P: 0.641). The results of the IVW analyses demonstrated that there is a causal association between T2DM and the risk of AD (OR: 1.237, 95% CI: 1.099–1.391, P: 0.0003). No evidence was found for the causal associations between MS and the risk of AD in the IVW method (OR: 0.984, 95% CI: 0.950–1.019, P: 0.381). There was no association between migraine and AD in the IVW method (OR: 20.264, 95% CI: 0.124–3287.978, P: 0.246). The detailed information of MR estimates for the exposures on AD risk is described in Table 2. Scatter plots and funnel plots from genetically predicted exposure on AD are shown in Figures 2–5.

**Table 2:** MR estimates for the exposures on AD risk.

Exposure	Methods	Odds ratio	95% CI	*p*
Type 1 diabetes	IVW	1.005	0.983–1.026	0.641
	WMR	0.989	0.960–1.019	0.506
	MR Egger	0.989	0.958–1.021	0.536
Type 2 diabetes	IVW	1.237	1.099–1.391	0.0003
	WMR	1.248	1.019–1.529	0.031
	MR Egger	1.304	0.973–1.746	0.081
Multiple sclerosis	IVW	0.984	0.950–1.019	0.381
	WMR	0.991	0.941–1.045	0.761
	MR Egger	0.843	0.939–1.052	0.994
Migraine	IVW	20.264	0.124–3287.978	0.246
	WMR	19.243	0.234–15824.467	0.387
	MR Egger	14.034	0.135–288.882	0.156

In order to evaluate the robustness of the abovementioned results, we conducted a sensitivity analysis, including Cochran’s Q test, MR-Egger intercept test, and MR-PRESSO global test. In the sensitivity analysis, the association patterns of most statistical models maintained directional consistency (Table 3). The leave-one-out analysis is shown in Supplementary Figures S1–S4.

**Table 3:** Sensitivity analysis of the causal association between exposure and the risk of AD.

Exposure	Cochran’s *Q* test		MR-Egger		MR-PRESSO value of *p*
	*Q* value	*p*	Intercept	*p*	
Type 1 diabetes	35.701	0.973	0.009	0.214	0.523
Type 2 diabetes	40.140	0.782	0.005	0.956	0.795
Multiple sclerosis	101.963	0.264	88.368	0.244	0.264
Migraine	12.367	0.336	0.086	0.103	0.392

## 4. Discussion

In this study, we performed several MR analyses from large consortia and genetic studies to individually investigate the causal relationship between TIDM, T2DM, MS, migraine, and AD. The result suggested that T2DM was causally associated with AD (OR: 1.237, 95% CI: 1.099–1.391, P: 0.0003 for IVW method; OR: 1.248, 95% CI: 1.019–1.529, P: 0.031 for WMR method). In addition, there was no causal link between TIDM, MS, migraine, and AD according to our results, which means that these chronic diseases would not be significantly associated with the development of AD. Perhaps, TIDM, MS, migraine, and AD are complicated diseases, and their development and progression would be caused by other factors.

Both T2DM and AD are common chronic diseases in the elderly, and there is a close relationship between them. Clinical studies have confirmed that T2DM increases the risk of cerebrovascular disease, impairs cognitive function, and may even lead to the development of AD. It has been reported that the prevalence of T2DM combined with AD is increasing, and the risk of AD in patients with T2DM is much higher than in patients without T2DM (Jia et al., 2020; Lorente et al., 2022; Cao et al., 2023). With the aging of the world population, the number of T2DM and AD patients in the world is increasing, and the prevention and treatment of T2DM and AD have become a global public health problem. Our two-sample MR study demonstrated a causal link between T2DM and AD risk, which is consistent with previous observational studies.

At present, exploring the high-risk molecular mechanism and new treatment methods for T2DM combined with AD is a hot spot of clinical research. Serum soluble apoptosis factor (sFas) and sFas ligand (sFasL) may be high-risk molecules for predicting T2DM combined with AD (Chi et al., 2022; Zheng et al., 2023). A Chinese study on T2DM combined with AD showed that the area under the curve (AUC) of serum sFas and sFasL in the diagnosis of T2DM combined with AD were 0.760 and 0.774, respectively, which suggested that both sFas and sFasL have certain diagnostic value for T2DM combined with AD. The AUC of serum sFas and sFasL in the combined diagnosis of T2DM combined with AD was 0.836, which indicated that the value of the combined diagnosis was higher (Damanik and Yunir, 2021). Therefore, it is necessary to closely monitor the serum sFas and sFasL levels of patients with T2DM for early AD prevention. Previous studies demonstrated that sFas and sFasL are not only related to apoptosis but also related to insulin resistance (Zheng et al., 2023). The occurrence of various central nervous system diseases is closely related to apoptosis, and sFas and sFasL play an important role in mediating apoptosis (Nortley et al., 2019). The reason for the increased serum sFas and sFasL levels in patients with T2DM combined with AD may be that hyperglycemia produces a variety of cytokines, which activate the sFas and sFasL systems, leading to increased expression of sFas and sFasL on the surface of the cells, causing apoptosis of central nervous system cells, and then altering cognitive functions (Nortley et al., 2019). Moreover, neuron-specific enolase (NSE) and phosphorylated tau (P-tau) in serum exosomes have also been considered indicators of cognitive impairment secondary to T2DM (Dove et al., 2021). Clinical studies involving 114 patients with T2DM suggested that the combination of NSE and P-tau in serum exosomes, which predicts the AUC of cognitive impairment in patients with T2DM, was 0.827 (Domínguez et al., 2014).

Several underlying mechanisms have been proposed to explain the increased risk of AD in patients with T2DM. First, the hyperglycemic state causes mitochondrial dysfunction, which eventually turns into neuronal apoptosis (Wang et al., 2022). Mitochondria, as an important organelle for cells to generate energy through aerobic respiration, play an important role in maintaining brain homeostasis and meeting the energy needs of neurons (Cabezas-Opazo et al., 2015). Hyperglycemia interferes with the balance of mitochondrial fusion and fission, affecting the number, shape, and function of mitochondria (Solanki et al., 2017; Wang et al., 2022). Abnormal mitochondrial function can generate a large amount of reactive oxygen species and enhance oxidative stress response (Zhang et al., 2022). Accumulating evidence suggests that mitochondria may be damaged to varying degrees in morphology, mitochondrial dynamics, and bioenergetics in the pathogenesis of AD, accelerating AD lesions in the brain (Cabezas-Opazo et al., 2015). Oxidative stress to mitochondria in neurons was increased in a study of a streptozotocin-induced diabetic mice model, and oxidative damage to brain mitochondria further led to impaired motor and memory behavioral functions (Solanki et al., 2017). Second, diabetic cerebrovascular damage could also explain the increased risk of AD in patients with T2DM. In the brains of patients with AD, about 8.1% of the cerebral blood vessels contracted due to Aβ deposition, resulting in a reduction of about 50% of the blood flow in the brain and a reduced energy supply. In patients with AD, the blood flow of gray matter decreased by approximately 42% (Kellar and Craft, 2020). Third, abnormal cholesterol in the state of type 2 diabetes can cause AD through the increase of Aβ. According to a perspective study, cholesterol plays a crucial role in the process of Aβ generation and aggregation. Another perspective study claimed that cholesterol can be changed by Aβ during neuronal dynamics, thereby promoting the subsequent development of cognitive impairment (Hanyu, 2019). Fourth, the central insulin signaling pathway is disrupted in patients with T2DM, and the impairment of insulin signaling can easily lead to a decrease in energy metabolism, specifically manifested in the decrease in ATP and glucose intake, the permeability and stability of nerve cells, and the negative impact of glucose metabolism disorders (Tamura et al., 2020). Finally, insulin resistance plays an important role in the overall occurrence and subsequent development of cognitive impairment in patients with T2DM. Patients with T2DM have insulin resistance both centrally and peripherally, and the insulin that breaks through the blood-brain barrier and is transported to the brain tissue is also greatly reduced, which ultimately damages energy, a stable form of glucose metabolism, and white matter fiber structure and function (Hanyu, 2019; Kellar and Craft, 2020; Tamura et al., 2020).

There was a causal link between T2DM and AD according to our MR analysis. Therefore, early identification and prediction of T2DM combined with AD is particularly important. In clinical practice, a few studies have discovered serum markers that can early predict the development of T2DM with cognitive impairment, such as sFas, sFasL, and P-tau (Dove et al., 2021; Chi et al., 2022; Zheng et al., 2023). We hope that, in the future, more scholars will pay attention to the study of T2DM combined with AD, actively search for the risk factors of T2DM combined with AD and explore the markers that can predict T2DM combined with AD, leading to early intervention and treatment of the disease.

This study includes several notable strengths. First, MR analysis, which can largely reduce the impact of environmental confounding factors and reverse causality, was used for the first time to explore the causal relationship between T1DM, T2DM, MS, and migraine and AD. Second, the selected SNPs accounted for a higher proportion of T1DM, T2DM, MS, and migraine. Furthermore, the large sample size of each MR analysis and the robust estimation effect of each instrumental variable guaranteed the statistical power of our study. Finally, the consistency of causality was confirmed by sensitivity analyses such as weighted median analysis, MR-Egger regression, MR-PRESSO, and leave-one-out analysis. However, several limitations should be considered when interpreting our findings. First, the participants in this study were all of European ancestry, which may limit the generalizability of our findings to other populations. Further studies are needed to validate our findings in populations of non-European ancestry. Second, the causal associations between AD and migraine subtypes (e.g., migraine with or without aura) were not further explored due to a lack of available GWAS with enough power for MR analysis. Third, we tried to figure out several confounder factors of AD, but there may be additional confounder factors that we did not figure out.

## 5. Conclusion

In conclusion, this two-sample MR study showed genetic evidence for the causal link between T2DM and AD. These findings highlight the significance of active monitoring and prevention of AD in patients with T2DM. We hope more scholars will pay attention to the study of T2DM combined with AD in the future, actively search for the risk factors of T2DM combined with AD and explore the markers that can predict T2DM combined with AD, leading to early intervention and treatment of the disease.

## Data availability statement

The original contributions presented in the study are included in the article/Supplementary material, further inquiries can be directed to the corresponding author.

## Author contributions

HX: conceptualization, resources, data curation, and writing – original draft. HX and LZ: methodology and formal analysis. HX and SL: software and investigation. HX: writing – review and editing. All authors contributed to the article and approved the submitted version.

## Conflict of interest

The authors declare that the research was conducted in the absence of any commercial or financial relationships that could be construed as a potential conflict of interest.

## Publisher’s note

All claims expressed in this article are solely those of the authors and do not necessarily represent those of their affiliated organizations, or those of the publisher, the editors and the reviewers. Any product that may be evaluated in this article, or claim that may be made by its manufacturer, is not guaranteed or endorsed by the publisher.
