# Supplementary material for: Unraveling the link: exploring the causal relationship between diabetes, multiple sclerosis, migraine, and Alzheimer’s disease through Mendelian randomization
Source: Front Neurosci. 2023 Aug 10;17:1233601. doi: 10.3389/fnins.2023.1233601 (PMC10488716; doi:10.3389/fnins.2023.1233601)
Supplement: Supplementary file 5 [file Data_Sheet_1.docx]

***Supplementary figures.***

**
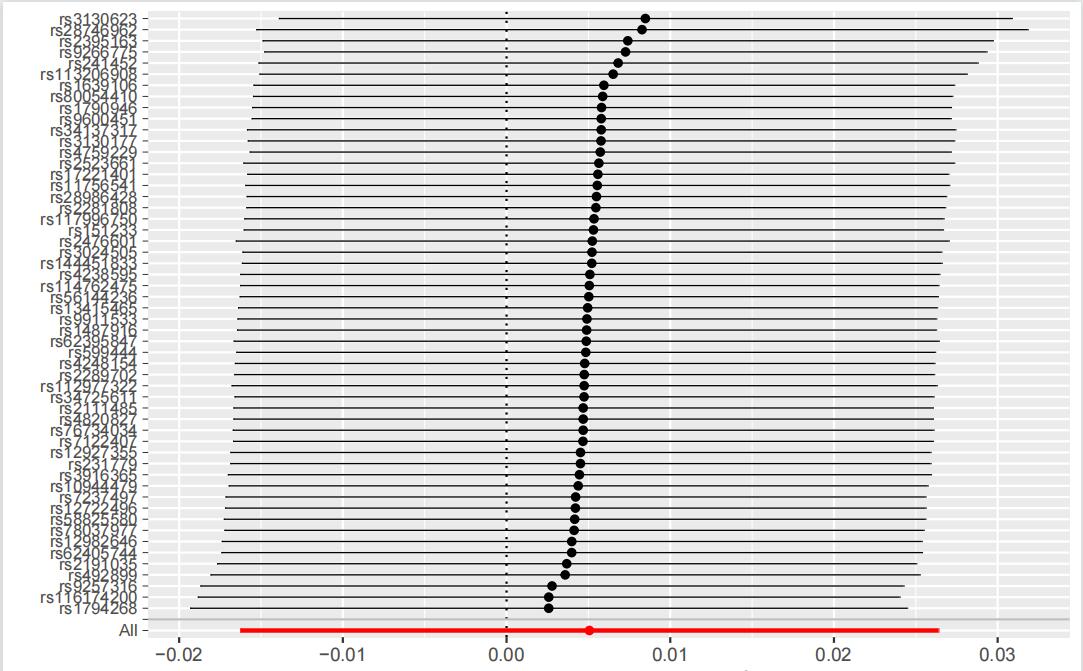
**

**Figure S1.** The leave-one-out analysis for genetically predicted assessed type 1 diabetes mellitus on Alzheimer’s disease


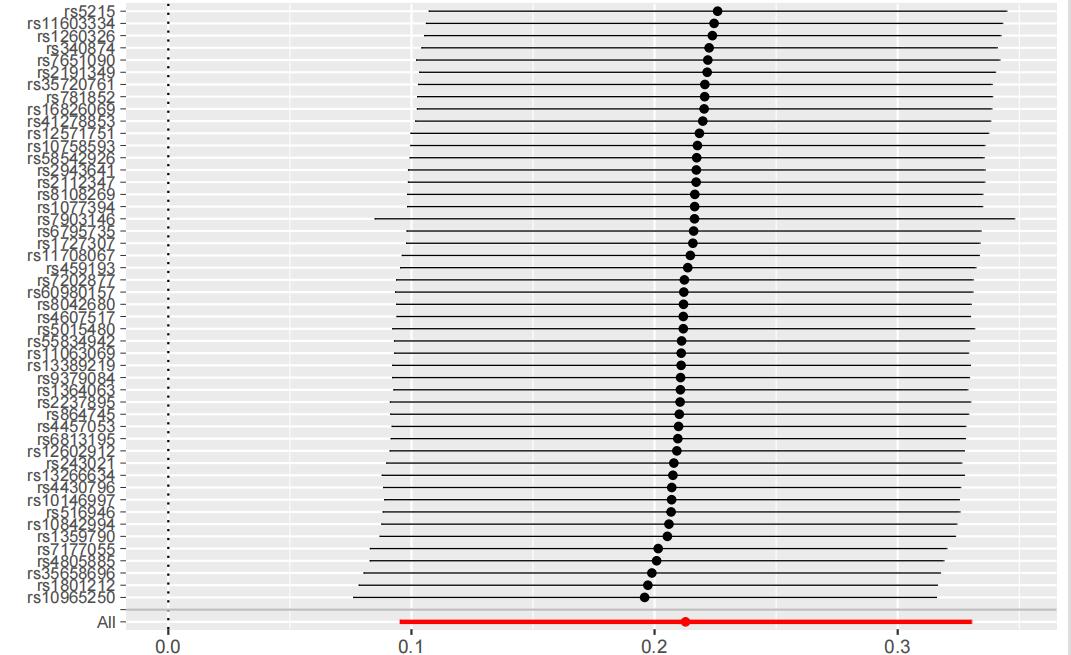


**Figure S2.** The leave-one-out analysis for genetically predicted assessed type 2 diabetes mellitus on Alzheimer’s disease


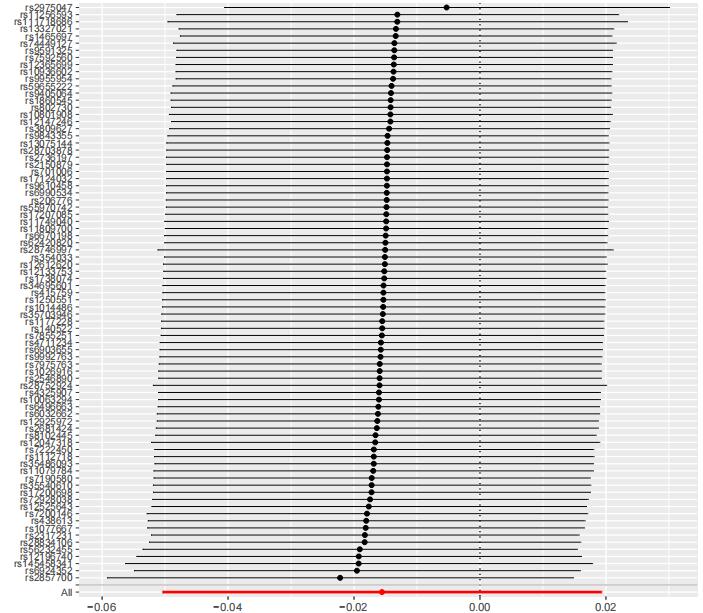


**Figure S3.** The leave-one-out analysis for genetically predicted assessed multiple sclerosis on Alzheimer’s disease


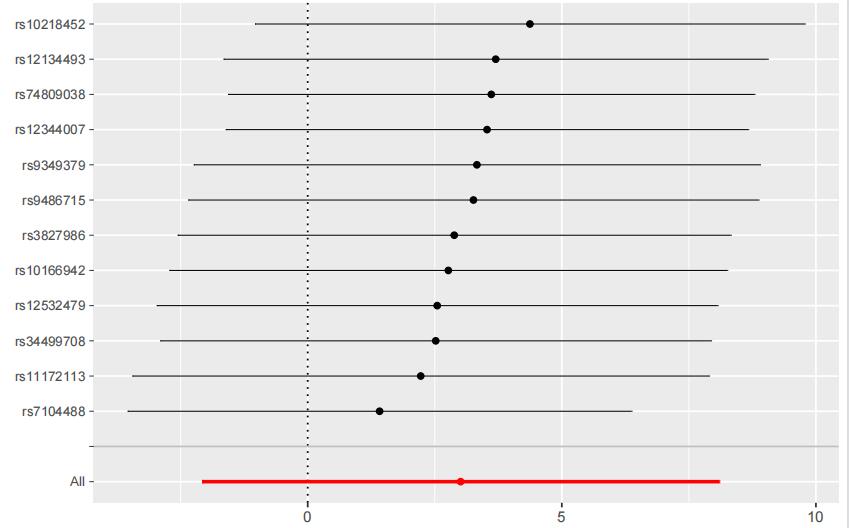


**Figure S4.** The leave-one-out analysis for genetically predicted assessed migraine on Alzheimer’s disease
